# Serum Fatty Acids Are Correlated with Inflammatory Cytokines in Ulcerative Colitis

**DOI:** 10.1371/journal.pone.0156387

**Published:** 2016-05-26

**Authors:** Dawn M. Wiese, Sara N. Horst, Caroline T. Brown, Margaret M. Allaman, Mallary E. Hodges, James C. Slaughter, Jennifer P. Druce, Dawn B. Beaulieu, David A. Schwartz, Keith T. Wilson, Lori A. Coburn

**Affiliations:** 1 Veterans Affairs Tennessee Valley Healthcare System, Nashville, Tennessee, United States of America; 2 Department of Medicine, Division of Gastroenterology, Hepatology, and Nutrition, Vanderbilt University Medical Center, Nashville, Tennessee, United States of America; 3 Department of Pathology, Microbiology, and Immunology, Vanderbilt University Medical Center, Nashville, Tennessee, United States of America; 4 Department of Cancer Biology, Vanderbilt University Medical Center, Nashville, Tennessee, United States of America; 5 Department of Medicine, Vanderbilt University Medical Center, Nashville, Tennessee, United States of America; 6 Department of Biostatistics, Vanderbilt University Medical Center, Nashville, Tennessee, United States of America; 7 University of Central Florida, College of Medicine, Orlando, Florida, United States of America; CWRU/UH Digestive Health Institute, UNITED STATES

## Abstract

**Background and Aims:**

Ulcerative colitis (UC) is associated with increased dietary intake of fat and n-6 polyunsaturated fatty acids (PUFA). Modification of fat metabolism may alter inflammation and disease severity. Our aim was to assess differences in dietary and serum fatty acid levels between control and UC subjects and associations with disease activity and inflammatory cytokines.

**Methods:**

Dietary histories, serum, and colonic tissue samples were prospectively collected from 137 UC subjects and 38 controls. Both histologic injury and the Mayo Disease Activity Index were assessed. Serum and tissue cytokines were measured by Luminex assay. Serum fatty acids were obtained by gas chromatography.

**Results:**

UC subjects had increased total fat and oleic acid (OA) intake, but decreased arachidonic acid (AA) intake vs controls. In serum, there was less percent saturated fatty acid (SFA) and AA, with higher monounsaturated fatty acids (MUFA), linoleic acid, OA, eicosapentaenoic acid (EPA), and docosapentaenoic acid (DPA) in UC. Tissue cytokine levels were directly correlated with SFA and inversely correlated with PUFA, EPA, and DPA in UC subjects, but not controls. 5-aminosalicylic acid therapy blunted these associations.

**Conclusions:**

In summary, we found differences in serum fatty acids in UC subjects that correlated with pro-inflammatory tissue cytokines. We propose that fatty acids may affect cytokine production and thus be immunomodulatory in UC.

## Introduction

Ulcerative colitis (UC), a subtype of inflammatory bowel disease (IBD), is limited to the mucosal layer of the colon and rectum. UC pathogenesis is thought to involve antigenic stimulation by enteric bacteria, fungi, or viruses in genetically susceptible individuals leading to a dysregulated chronic inflammatory state [1, 2]. In UC, alteration in both humoral immunity (via IgG1 and IgG3) and cellular immunity (T-cell mediated and innate immunity) has been shown [3, 4]. This inflammatory state is marked by increased eicosanoids such as prostaglandin E_2_ and leukotriene B_4_ [5–8], which are derived from polyunsaturated fatty acid (PUFA) metabolism and are reduced by 5-aminosalicylic acid (5-ASA) agents, which are a major component of UC treatment [7, 9].

Because of the potential role of fatty acids in inflammation, fatty acid profile detection and manipulation have been an area of interest in UC. Epidemiologic studies have shown an increased prevalence of IBD that correlates with increased animal fat and n-6 PUFA intake [10]. Large cohort studies have identified increased linoleic acid (LA) and arachidonic acid (AA) intake in UC patients [11, 12] as well as increased AA in adipose tissue [13]. Clinical studies have shown mixed results with regards to fatty acid composition and manipulation. It has been hypothesized that IBD patients would have decreased blood and tissue PUFA, specifically n-3 PUFA, due to the increased inflammatory state. However, several studies have shown increased PUFA in blood samples with higher pro-inflammatory, or n-6 pathway, metabolites [14, 15]. PUFA concentrations were higher in all UC subjects, but levels decreased with greater disease activity without reaching the levels of controls [15]. Plasma fatty acid composition changes persist despite lack of disease activity, even after colectomy [16], suggesting an intrinsic alteration in fatty acid profiles that is independent of disease activity. These changes have been observed at the tissue level as well with higher percent saturated fatty acids (SFA) and PUFA in UC subjects’ colon tissue versus controls [17]. These changes correlated with endoscopic and histologic disease activity [18].

The mechanism by which fatty acids influence IBD is not fully understood, but it has been suggested that n-6 PUFA promote pro-inflammatory cytokines via metabolism of AA [19, 20]. In addition, n-3 PUFA have anti-inflammatory properties including displacement of AA from the cell membrane with resultant decreased derivatives, altered cell membrane fluidity and protein binding capability, and inhibition of NF-κB and its nuclear targets [21]. A recent study identified specific AA metabolites (prostaglandins E_2_ and D_2_, thromboxane B_2_, and hydroperoxyeicosatetraenoic acids (HETE) products) as elevated in UC colonic tissue and predictive of colonic inflammation [22].

Previous research in our UC cohort identified elevations in serum eotaxin-1 and G-CSF as well as tissue eotaxin-1, G-CSF, IP-10, IL-6, TNF-α, IL-17, MCP-1, MIP-1α, MIP-1β, IL-1α, IL-1β, IL-1RA, and IL-8 in UC subjects [23]. These differences were more significant in active UC and, in the case of eotaxin-1, persisted at all levels of disease activity. Our current study aimed to: 1) investigate fatty acid dietary intake and serum fatty acid composition patterns in UC and control subjects, and to identify whether these patterns are associated with disease activity; and 2) determine if there is an association of serum fatty acid composition with the serum and tissue inflammatory markers associated with active UC that we previously identified in our cohort.

## Materials and Methods

### Ethical Considerations

The study protocol was approved by the Vanderbilt University Institutional Review Board. Written informed consent was obtained from all subjects for: analyses of demographics, medical and dietary histories, as well as serum and tissue biopsies obtained at colonoscopy. These studies were registered on clinical trials.gov as “Effects of L-Arginine in Colitis and Colon Cancer”, identifier NCT01091558.

### Patients

Samples were obtained from a previously collected cohort of control and UC subjects [23]. Subjects were prospectively recruited in the IBD clinic or endoscopy unit at Vanderbilt University Medical Center prior to outpatient colonoscopy for colorectal cancer screening, evaluation for possible IBD, or UC surveillance purposes between September 2009 and September 2011. All subjects underwent overnight fast and received polyethylene glycol electrolyte solution for bowel preparation prior to colonoscopy. As described [23], all subjects consented to serum collection as well as tissue biopsies for research purposes in four colonic segments (ascending, transverse, descending, and rectum) at the time of colonoscopy. Study serum and tissue biopsies were snap frozen with dry ice, and then stored at −80°C.

Surveillance biopsies from UC subjects were reviewed by the Vanderbilt University Department of Pathology and graded as: normal, quiescent, mild, moderate, or severe activity. The Mayo Disease Activity Index (DAI) was determined for UC subjects at the time of colonoscopy by standard measures (0–12 scale) [9, 23]. Endoscopic severity and the physician’s global assessment for the DAI were determined by IBD specialists (D.A.S.; D.B.B.; S.N.H.) as follows: normal, mild disease (erythema, decreased vascular pattern, mild friability), moderate disease (marked erythema, lack of vascular pattern, friability, erosions), or severe disease (spontaneous bleeding, ulceration). Patients who were pregnant, had known coagulopathy or bleeding disorders, known renal or hepatic impairment, prior organ transplant, or were unable to give informed consent were excluded. Further details regarding inclusion and exclusion have been described [23].

### Dietary Intake

We used the average of 3 different 24-hour diet recall interviews to assess dietary intake. A trained research coordinator interviewed patients on 3 occasions to obtain separate 24-hour dietary intake histories using the Nutrient Data System for Research software (U. of MN) [24]. The first interview was done within 1 week of the study colonoscopy, but did not include the day of or prior to colonoscopy, as fasting and colonoscopy preparation would affect dietary intake. The second and third interviews were completed based on patient availability, and we attempted to sample both weekdays and weekends. The average daily nutrient intake was calculated based on each patient’s set of interviews and used for analysis. Dietary fat intake was analyzed as the absolute amount in grams, as well as a percent of the total kilocalorie intake.

### Serum Fatty Acids

Serum fatty acids were analyzed by the Vanderbilt Hormone Assay Core laboratory. Serum lipids were extracted using the Folch et al method [25]. The extracts were filtered, and lipids recovered in the chloroform phase. Individual lipid classes were separated by thin layer chromatography using Silica Gel 60 A plates developed in petroleum ether, ethyl ether, acetic acid (80:20:1), and visualized by rhodamine 6G. Phospholipids, diglycerides, triglycerides, and cholesteryl esters were scraped from the plates and methylated using BF3/methanol as Morrison and Smith described [26]. The methylated fatty acids were extracted and analyzed by gas chromatography. Gas chromatographic analyses were carried out on an Agilent 7890A gas chromatograph equipped with flame ionization detectors, and a capillary column (SP2380, 0.25 mm x 30 m, 0.25 μm film, Supelco, Bellefonte, PA). The carrier gas was helium. The oven temperature was programmed from 160°C to 230°C at 4°C/min. Fatty acid methyl esters were identified by comparing the retention times to those of known standards. Inclusion of lipid standards with odd chain fatty acids permitted quantitation of the lipid amounts in the sample. Dipentadecanoyl phosphatidylcholine (C15:0), diheptadecanoin (C17:0), trieicosenoin (C20:1), and cholesteryl eicosenoate (C20:1) were used as standards. Output was expressed as percent of total phospholipid composition.

### Serum and Tissue Cytokines

Our methods for serum and tissue cytokine measurement have been described in detail [23]. Briefly, samples were assessed using Luminex technology with Milliplex MAP (Millipore, Billerica, MA) multiplex magnetic bead-based antibody detection kits according to the manufacturer’s protocols. Tissues were lysed in radioimmunoprecipitation assay (RIPA) buffer with a mortar and pestle-type rotary homogenizer, and assayed on a FLEXMAP 3D machine. The tissue lysate protein concentration was measured using the bicinchoninic acid (BCA) method.

### Statistical Analysis

Data are expressed as mean ± SD or number (%). Outlier testing was done on all data using the Grubbs method, also called the extreme studentized deviate method [27]. For 2 group comparisons, an unpaired Mann-Whitney U test was completed [28]. Data with more than 2 groups was analyzed with a Kruskal-Wallis test and if *P* < 0.05, then pairwise comparisons were made with the Mann-Whitney U test. Categorical data was analyzed with a Pearson's χ^2^ test. Correlations were determined using Spearman’s rank correlation and presented with the correlation coefficient and the *P* value. We used logistic regression to estimate the association between UC status (versus control) and a 1-standard deviation increase in each fatty acid both unadjusted and adjusted for age, BMI, or gender [29]. We estimated separate models for each of the adjustment variables due to the limited number of events [30]. A 10% change in the odd ratios for the fatty acid (comparing adjusted to unadjusted) was considered evidence of confounding. Logistic regression results are summarized using odds ratio (OR) with the 95% confidence interval (95% CI). When multiple comparisons were performed for associations with tissue cytokines, we considered *P* < 0.01 as significant. Statistical analyses were completed with STATA v 13.1 (College Station, TX).

## Results

### Patient Characteristics

In total, 137 UC subjects and 38 control subjects were included in the original cohort. We had serum samples available for fatty acid analysis on 23 control subjects and 101 UC subjects of which 35 had quiescent disease, 29 had mild disease, 22 had moderate disease, and 15 had severe disease based on histology. The baseline characteristics of subjects that did or did not have serum fatty acid profiles obtained were not different (data not shown). Of subjects included in this study, the UC subjects were significantly younger than the control subjects, but there was no significant difference in gender distribution or BMI (Table 1). 95% of the UC subjects were on some therapy for their UC, and 66/101 (65.3%) had active disease determined by histology. Subjects with quiescent disease were more likely to be on anti-TNF-α medications, but had similar rates of 5-ASA, steroid, and immunomodulator use compared to those with active disease (Table 1).

**Table 1:** Patient characteristics.

	Control (*n* = 23)	Quiescent UC (*n* = 35)	Active UC (*n* = 66)
Age, mean ± SD	51.3 ± 13.8	42.6 ± 14.2^a^	42.7 ± 15.1^a^
Gender	39.1% Male	42.9% Male	45.5% Male
Body Mass Index, mean ± SD	26.4 ± 9.5	27.6 ± 5.9	27.1 ± 7.3
Tobacco Use	2 (8.7%)	3 (8.6%)	4 (6.1%)
Any IBD Therapy	1 (4.4%)	34 (97.1%)	62 (93.9%)
Any 5-ASA	1 (4.4%)^e^	28 (80.0%)^c^	54 (81.8%)^c^
Corticosteroids	0	4 (11.4%)	11 (16.7%)^a^
Immunomodulators	0	13 (37.1%)^b^	19 (28.8%)^b^
Anti-TNF-α	0	12 (25.3%)^b^	10 (15.2%)^a^^,^^d^
Completed Dietary Interview	17 (73.9%)	24 (68.6%)	49 (74.2%)

UC status determined by histologic activity.

^a^*P* < 0.05,

^b^*P* < 0.01,

^c^*P* < 0.001 versus control;

^d^*P* < 0.05 versus quiescent UC.

^e^This patient was started on therapy for presumed UC but was subsequently deemed to have irritable bowel syndrome.

Age and body mass index statistics were assessed by the Kruskal-Wallis test followed by the Mann-Whitney U test. Categorical data were analyzed using the Pearson’s χ^2^ test.

### Dietary Intake

Overall, 72.6% of the subjects completed the dietary intake assessment; the response rate was not different among the subgroups (Table 1). Baseline characteristics among subjects that did or did not complete the dietary assessment were not different (data not shown). The mean number of days between serum collection and completion of all dietary recall interviews was 33, with a standard deviation of 35.1 days. Total dietary intake of fat and percent fat was higher in the UC subjects versus controls (Table 2). Intake of total kilocalories, SFA, monounsaturated fatty acids (MUFA), and PUFA was modestly increased in the UC subjects but did not reach statistical significance. Intake of AA and oleic acid (OA) was significantly higher in UC subjects (Table 2). Fatty acid intake was not associated with disease activity by histology or DAI (data not shown). BMI was not associated with total energy intake (control, *P* = 0.547; UC, *P* = 0.521) or fat intake (control, *P* = 0.859; UC, *P* = 0.156). There was a trend towards lower fat intake in UC subjects on steroid therapy (64.45 ± 23.07 g/day on steroids versus 54.38 ± 26.25 g/day not on steroids; *P* = 0.077), but no other associations with fat intake and medication use were observed.

**Table 2:** Dietary Intake.

	Control (*n* = 17)	UC (*n* = 73)	*P*
Energy (Kcal)	1556.13 ± 466.57	1739.76 ± 653.01	0.255
Fat (g)	53.17 ± 33.08	62.35 ± 23.93	0.049
Fat (% total Kcal)	32.11 ± 10.89	36.70 ± 7.13	0.046
SFA (g)	17.51 ± 10.69	20.92 ± 8.75	0.102
PUFA (g)	11.84 ± 8.35	13.54 ± 6.72	0.122
MUFA (g)	19.29 ± 12.38	22.86 ± 9.18	0.063
n-3 (g)	1.13 ± 0.55	1.32 ± 0.63	0.292
n-6 (g)	10.65 ± 7.88	12.12 ± 6.25	0.135
n-3/n-6	0.11 ± 0.02	0.11 ± 0.04	0.722
AA (g)	0.08 ± 0.05	0.13 ± 0.08	0.040
Linoleic (g)	10.56 ± 7.85	12.021 ± 6.25	0.147
α-Linolenic (g)	1.04 ± 0.51	1.19 ± 0.56	0.428
OA (g)	17.95 ± 11.61	21.41 ± 8.84	0.048

Data are expressed as mean ± SD in average grams/day or average percent of total kilocalorie intake. Mann-Whitney U test was performed.

### Serum Fatty Acids Are Altered in UC Subjects Compared to Controls

Serum fatty acid profiles were significantly different between UC and controls subjects (Table 3). When all UC subjects were considered, they had significantly lower percent SFA, higher percent MUFA, and a trend towards higher percent total PUFA and n-3 PUFA. n-6 PUFA were similar between control and UC subjects. The eicosapentaenoic acid (EPA) docosahexaenoic acid (DHA)/AA ratio and percent OA and LA were significantly increased in UC subjects, while percent AA was decreased. The odds of having UC were lower in patients with lower percent SFA or AA, whereas the odds were increased with increased serum percent LA, DPA, and EPA (Table 3). In multivariable models where we adjusted for age, most adjusted odds ratios were within 10% of the unadjusted odds ratios (Table 3). The exception was with EPA where the adjusted OR was significantly increased. There were no significant changes in the OR when adjusting for gender or BMI (results not shown).

**Table 3:** Serum fatty acid percentages in control and UC subjects with unadjusted and age adjusted odds ratios.

			Unadjusted	Age Adjusted
	Control (*n* = 23)	UC (*n* = 101)	OR (95% CI)	OR (95% CI)
Total fat (μg/ml)	1434.73 ± 355.48	1307.37 ± 373.90	0.72 (0.46, 1.11)	0.99 (0.99, 1.00)
%SFA	46.51 ± 1.80	44.92 ± 2.40**	0.50 (0.30, 0.81)	0.46 (0.27, 0.77)
%PUFA	44.90 ± 1.89	45.84 ± 2.54	1.46 (0.93, 2.29)	1.63 (1.01, 2.64)
%MUFA	8.58 ± 1.65	9.24 ± 1.34**	1.74 (0.99, 3.04)	1.58 (0.91, 2.77)
%n-3	5.07 ± 1.80	5.70 ± 1.83	1.44 (0.89, 2.34)	1.59 (0.95, 2.70)
%n-6	39.83 ± 2.32	40.13 ± 2.20	1.15 (0.73, 1.81)	1.19 (0.75, 1.88)
n-3/n-6	0.13 ± 0.05	0.14 ± 0.05	1.41 (0.85, 2.35)	1.56 (0.91, 2.71)
EPA+DHA/AA	0.28 ± 0.09	0.36 ± 0.15**	2.37 (1.19, 4.71)	2.44 (1.21, 4.93)
%AA	15.38 ± 2.58	13.28 ± 2.52***	0.42 (0.25, 0.68)	0.46 (0.27, 0.76)
%LA	18.06 ± 3.06	20.12 ± 2.51**	2.19 (1.32, 3.63)	2.07 (1.24, 3.46)
%OA	7.06 ± 1.22	7.73 ± 1.10**	1.98 (1.11, 3.49)	1.81 (1.03, 3.20)
%EPA	0.63 ± 0.50	0.98 ± 0.60**	1.94 (1.17, 3.23)	2.16 (1.25, 3.74)
%DPA	0.72 ± 0.54	1.10 ± 0.43**	2.04 (1.32, 3.16)	2.24 (1.39, 3.62)
%DHA	3.70 ± 1.19	3.66 ± 1.30	0.97 (0.61, 1.52)	1.04 (0.64, 1.70)

Data are presented as mean ± SD. Mann-Whitney U test was performed for mean comparison of UC vs controls.

*P < 0.05,

**P < 0.01,

***P < 0.001.

Logistic regression of probability of UC was performed for unadjusted and age-adjusted analysis and displayed as Odds Ratio (OR) and 95% confidence interval (95% CI).

As shown in Table 4, the differences in percent serum SFA, PUFA, EPA+DHA/AA, EPA, and docosapentaenoic acid (DPA) were greater between histologically quiescent UC subjects versus controls; while AA, LA, and OA showed similar differences from controls in both active and quiescent UC. When disease activity was defined by DAI, serum fatty acids remained altered in patients with inactive or active disease versus controls; however, there were no differences between inactive and active disease (results not shown). The odds of having active (versus inactive) disease were not associated with serum fatty acids in unadjusted or adjusted models (results not shown).

**Table 4:** Serum fatty acid percentages by histologic disease activity.

	Control (*n* = 23)	Quiescent UC (*n* = 35)	Active UC (*n* = 66)
Total fat (μg/ml)	1434.73 ± 355.48	1353.51 ± 408.83	1282.90 ± 354.81
%SFA	46.51 ± 1.80	44.32 ± 1.96***	45.24 ± 2.55
%PUFA	44.90 ± 1.89	46.53 ± 2.06**	45.47 ± 2.71
%MUFA	8.58 ± 1.65	9.14 ± 1.10	9.28 ± 1.45
%n-3	5.07 ± 1.80	6.08 ± 1.60	5.50 ± 1.92
%n-6	39.83 ± 2.32	40.45 ± 2.23	39.97 ± 2.19
n-3/n-6	0.13 ± 0.05	0.15 ± 0.05	0.13 ± 0.05
EPA+DHA/AA	0.28 ± 0.09	0.39 ± 0.15**	0.34 ± 0.14
%AA	15.38 ± 2.58	13.05 ± 2.47***	13.37 ± 2.55***
%LA	18.06 ± 3.06	20.32 ± 2.59**	19.98 ± 2.49**
%OA	7.06 ± 1.22	7.65 ± 0.86**	7.79 ± 1.22**
%EPA	0.63 ± 0.50	1.06 ± 0.57**	0.94 ± 0.62
%DPA	0.72 ± 0.54	1.17 ± 0.36**	1.05 ± 0.46
%DHA	3.70 ± 1.19	3.84 ± 1.25	3.57 ± 1.32

UC activity determined by histology. Data are presented as mean ± SD. If *P* < 0.05 by Kruskal-Wallis test, Mann-Whitney U test was performed.**P < 0.01 and ***P < 0.001 compared to the control group.

Dietary intake did not correlate with the measured fatty acids in Table 4 in UC or control subjects (all *P* > 0.05, data not shown). Therefore, the alterations in serum fatty acids are unlikely to be due simply to alterations in dietary intake.

### Detailed Analysis of Serum Fatty Acids by Histologic Disease Activity

**Fig 1:**
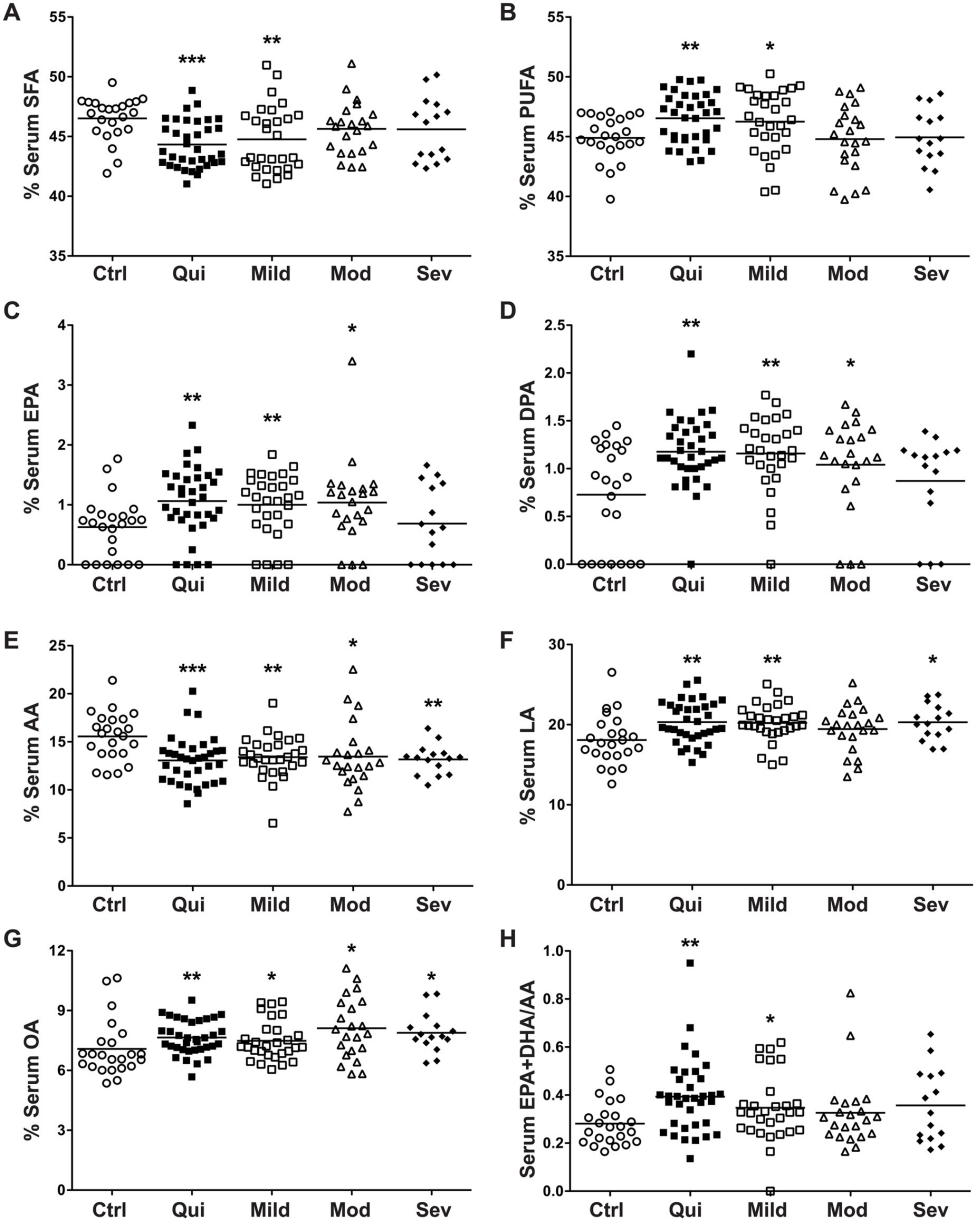
Detailed analysis of serum fatty acids by histologic disease activity. Blood was obtained from each subject and processed within 30 min to obtain serum. Serum fatty acid levels were measured as described in the Methods. Disease categories were based on maximal tissue histopathology. *n* = 23 for control (Ctrl) and *n* = 101 for UC subjects. *P < 0.05, **P < 0.01, ***P < 0.001 versus control.

When UC subjects were categorized by histologic disease severity (quiescent, mild, moderate, severe), several serum fatty acid levels were altered versus control subjects. Serum percent SFA was decreased while percent PUFA and the EPA+DHA/AA ratio were increased in both quiescent and mild UC (Fig 1A, 1B and 1H). Serum percent EPA and DPA were increased (Fig 1C and 1D) in quiescent, mild, and moderate UC versus control subjects. Serum percent AA was decreased at all levels of UC disease severity (Fig 1E), while serum percent OA was increased at all levels (Fig 1G). Serum percent LA was increased in quiescent, mild, and severe UC (Fig 1F), while the modest increase in moderate UC was not statistically significant. Taken together, when comparing the serum fatty acids in UC versus control subjects, the alterations were most apparent in quiescent or mildly active UC. No differences were detected between levels of histologic severity in UC subjects. In addition, there were no correlations between any of the serum fatty acids and clinical activity defined by DAI.

### Serum Eotaxin-1 and G-CSF Are Not Correlated With Serum Fatty Acid Levels in UC

In our original cohort, we reported significant increases in both serum eotaxin-1 and G-CSF levels in patients with active UC versus control subjects [23]. In the current subset with serum fatty acid levels, serum percent PUFA was inversely correlated with eotaxin-1 levels in the control subjects, but this correlation was lost in the UC subjects (r = –0.44, *P* = 0.031 in controls; r = 0.02, *P* = 0.989 in UC). No other significant correlations were identified between the serum fatty acid levels and either eotaxin-1 or G-CSF.

### Multiple Tissue Cytokines Are Correlated With Serum Fatty Acid Levels in UC

**Fig 2:**
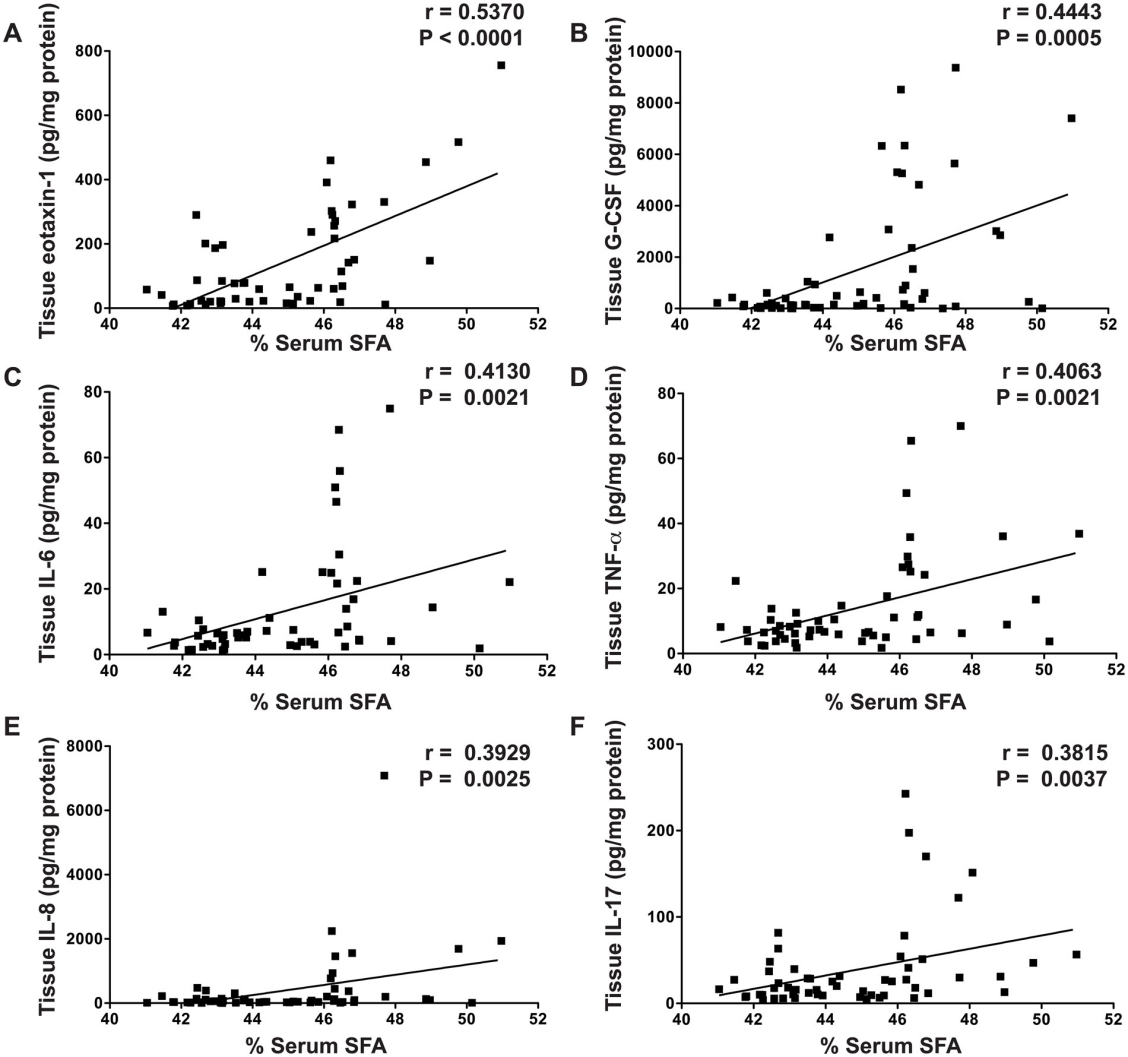
Tissue cytokines are directly correlated with serum percent SFA. Snap frozen colonic biopsies were lysed and cytokine/chemokine levels were measured by Luminex technology, with each sample corrected for tissue lysate protein concentration, as described in the Methods. Blood was obtained from each subject and processed within 30 min to obtain serum. Serum fatty acid levels were measured as described in the Methods. The Spearman correlation coefficient (r) and the associated *P* value are shown. *n* = 58 for UC subjects. There were no significant correlations in control subjects (data not shown).

**Fig 3:**
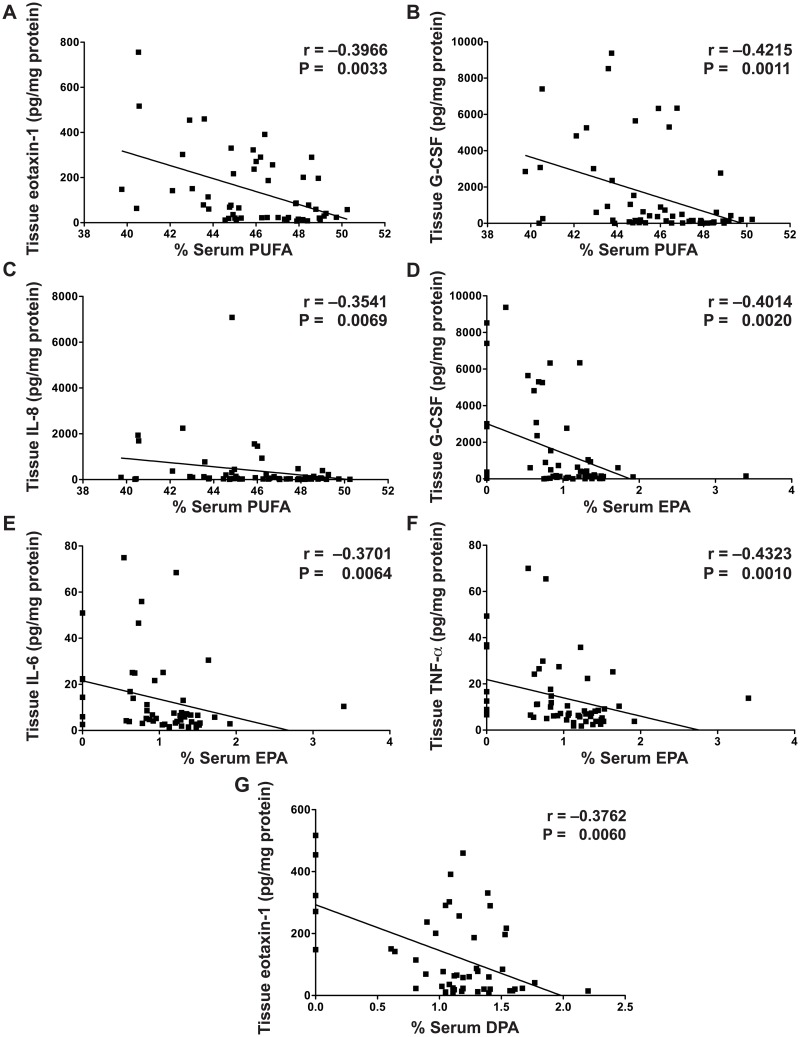
Tissue cytokines are inversely correlated with percent serum PUFA, EPA, and DPA. Snap frozen colonic biopsies were lysed and cytokine/chemokine levels were measured by Luminex technology, with each sample corrected for tissue lysate protein concentration, as described in the Methods. Blood was obtained from each subject and processed within 30 min to obtain serum. Serum fatty acid levels were measured as described in the Methods. The Spearman correlation coefficient (r) and the associated *P* value are shown. (A–C) Inverse correlations with percent serum PUFA. (D–F) Inverse correlations with percent serum EPA. (G) Inverse correlation with percent serum DPA. *n* = 58 for UC subjects. There were no significant correlations in control subjects (data not shown).

Previously, we identified that 13 out of 42 cytokines tested (eotaxin-1, G-CSF, IP-10, IL-6, TNF-α, IL-17, MCP-1, MIP-1α, MIP-1β, IL-1α, IL-1β, IL-1RA, IL-8) were significantly increased in the colonic tissue of active UC subjects [23]. We have now identified multiple associations between these tissue cytokines and the serum fatty acid levels in UC subjects that were not present in control subjects. Eotaxin-1, G-CSF, IL-6, TNF-α, IL-8, and IL-17 levels were directly correlated with serum percent SFA in UC (Fig 2A–2F). Eotaxin-1, G-CSF, and IL-8 were inversely correlated with percent PUFA (Fig 3A–3C); G-CSF, IL-6, and TNF-α were inversely correlated with percent EPA (Fig 3D–3F); and eotaxin-1 was inversely correlated with percent DPA (Fig 3G) in UC. There were no further correlations between fatty acids and tissue cytokines (S1 Table). No serum fatty acids were associated with tissue cytokines in control subjects (data not shown).

### UC Medications Have an Effect on Serum Fatty Acids

The serum fatty acid composition in UC subjects on immunomodulators, biologics, or 5-ASA agents was not different. Serum percent PUFA was higher in those not on steroids (46.02% ± 2.62%, versus 44.79% ± 1.77% on steroids, *P* = 0.049). In patients on biologics or steroid therapy, the association of their tissue cytokines with serum percent PUFA or percent SFA was lost (data not shown). The relationship between serum percent PUFA and percent SFA was stronger in UC subjects not taking a 5-ASA versus those taking a 5-ASA (Table 5).

**Table 5:** Effect of 5-ASA on association with tissue cytokines and serum fatty acids.

	No 5-ASA use (*n* = 9)		Current 5-ASA use (*n* = 49)	
	r	*P*	r	*P*
**%SFA**				
Eotaxin-1	1.00	<0.001	0.45	0.002
G-CSF	0.63	0.060	0.38	0.008
IL-6	0.95	<0.001	0.30	0.047
TNF-α	0.89	0.007	0.31	0.034
IL-8	0.88	0.004	0.30	0.036
IL-17	0.83	0.005	0.30	0.042
**%PUFA**				
Eotaxin-1	−0.93	0.003	−0.38	0.010
G-CSF	−0.75	0.020	−0.37	0.009
IL-8	−0.81	0.015	−0.34	0.018
**%EPA**				
G-CSF	−0.48	0.188	−0.34	0.019
IL-6	−0.81	0.015	−0.24	0.107
TNF-α	−0.86	0.014	−0.35	0.016
**%DPA**				
Eotaxin-1	−0.67	0.102	−0.30	0.045

Serum fatty acid and tissue cytokine associations identified as significant (Fig 3) were reanalyzed by use of 5-ASA in UC subjects only. Spearman correlation coefficient (r) and the associated *P* value are shown.

## Discussion

Overall fat intake in UC subjects was increased but was not associated with disease severity by histology or DAI. The increased AA intake seen in our UC subjects (Table 2) is similar to the findings of a large epidemiologic study from the Netherlands; however, that study identified lower OA intake in UC, whereas we identified the opposite relationship (Table 2) [12]. We did not find any other previously identified differences in fat intake. While previous studies have shown serum or plasma fatty acids to be accurate markers of short-term fatty acid intake [31, 32], we did not find associations between reported dietary intake and serum fatty acid levels in control or UC subjects. Contributing factors to this lack of association could have included timing of the serum fatty acid collection and the dietary interviews, limitations of diet history recall, or limitations in the dietary software capabilities.

The current study demonstrated significant differences in percent serum fatty acids between UC and controls (Tables 3 and 4). These results support an earlier study suggesting an upregulation of PUFA and the anti-inflammatory n-3 pathway in IBD that decreased with greater disease activity [15]. This is an interesting concept suggesting a metabolic phenomenon in IBD leading to altered fatty acid synthesis. As disease activity increases, the consumption of PUFA may increase in parallel, consistent with the pathophysiology of IBD and eicosanoid metabolism.

We were further interested in estimating the associations of serum fatty acids with the diagnosis of UC and the impact of age, gender and BMI on these associations. Using separate models adjusting for age, gender, or BMI, lower serum percent SFA and AA were associated with decreased odds of UC, while having higher LA, EPA, and DHA were associated with increased odds of having UC. Age was a potential confounder in some models; adjusting for age significantly increased the odds ratios for EPA only (Table 3). We found no evidence of an association between fatty acid percentage and having active UC (data not shown) in the adjusted or unadjusted model. However, these analyses were limited by the small sample size of patients with active UC.

Percent serum SFA and PUFA were significantly correlated with tissue cytokine levels in UC subjects only. The direct correlation with SFA and inverse correlation with PUFA suggests a pro-inflammatory role of SFA and an anti-inflammatory role for PUFA. Combining this with our data that UC subjects with active disease have higher percent serum SFA and a trend towards lower percent serum PUFA would support manipulation of fatty acid composition through diet or medication to modify cytokine production.

Our subgroups for medication analysis were limited by the lack of treatment naïve patients; however, we did find that UC subjects on biologics or immunomodulators lost the relationship between serum fatty acids and cytokine levels suggesting that these therapies blunt the influence of fatty acids on the inflammatory process. Similarly, those that were not on a 5-ASA had the strongest relationship between tissue cytokines and fatty acid composition. This suggests a relationship between fatty acid metabolism to eicosanoids and inflammatory effects on colonic tissue, which is no longer apparent with inhibitors of eicosanoid synthesis (i.e., 5-ASAs). Maximizing 5-ASA therapy in UC subjects that have a more pro-inflammatory fatty acid profile may be beneficial.

This study is limited by the lack of tissue fatty acid profiles. We did not have sufficient tissue samples remaining from the original study cohort to measure these levels. Previous studies have identified higher SFA and PUFA as well as AA, DPA, and DHA in inflamed UC tissue versus controls and uninflamed UC tissue [17, 18]. In addition, we were limited by the number of subjects in some of our subgroup analyses, such as a lack of treatment naïve subjects, or those with more severe disease. There are also limitations of the dietary intake assessment, as we did not have dietary assessment in all study subjects.

In summary, patients in our UC cohort had altered serum fatty acid profiles that may not be completely explained by dietary intake and that correlated with pro-inflammatory tissue cytokines. We propose a mechanism where PUFA may be upregulated in quiescent disease and consumed as disease activity increases. This correlates with local tissue cytokines and supports a role of PUFA in controlling the severity of inflammation. Manipulation of the fatty acid profile to increase PUFA and decrease SFA could play a useful role in controlling inflammation.

## Supporting Information

S1 Table Non-significant correlations of serum fatty acids with tissue cytokines in UC subjects. Percent serum fatty acid and tissue cytokine levels were analyzed by Spearman correlation. Presented is the Spearman correlation coefficient (r) and associated *P* value. Significant associations can be found in Figs 2 and 3.
(DOCX)
