# Supplementary material for: Serum Fatty Acids Are Correlated with Inflammatory Cytokines in Ulcerative Colitis
Source: PLoS One. 2016 May 26;11(5):e0156387. doi: 10.1371/journal.pone.0156387 (PMC4882051; doi:10.1371/journal.pone.0156387)
Supplement: S1 Table — Percent serum fatty acid and tissue cytokine levels were analyzed by Spearman correlation. Presented is the Spearman correlation coefficient (r) and associated P value. Significant associations can be found in Figs 2 and 3. (DOCX) [file pone.0156387.s001.docx]

**S1 Table: Non-significant correlations of serum fatty acids with tissue cytokines in UC subjects**

|  | **UC**  (*n* = 58) | |
| --- | --- | --- |
|  | **r** | ***P*** |
| **%SFA** | | |
| Il-1β | 0.31 | 0.023 |
| Il-1RA | 0.21 | 0.115 |
| Il-1α | 0.10 | 0.475 |
| MIP-1β | 0.34 | 0.011 |
| MIP-1α | 0.22 | 0.123 |
| MCP-1 | 0.23 | 0.084 |
| Il-7 | 0.01 | 0.927 |
| IP-10 | 0.19 | 0.150 |
| **%PUFA** | | |
| IL-6 | –0.30 | 0.027 |
| TNF-α | –0.28 | 0.042 |
| Il-1β | –0.15 | 0.295 |
| Il-1RA | –0.26 | 0.052 |
| Il-1α | –0.01 | 0.958 |
| MIP-1β | –0.25 | 0.063 |
| MIP-1α | –0.15 | 0.274 |
| MCP-1 | –0.15 | 0.270 |
| Il-7 | 0.01 | 0.963 |
| IP-10 | –0.13 | 0.331 |
| **%EPA** | | |
| Eotaxin-1 | –0.31 | 0.022 |
| IL-8 | –0.31 | 0.019 |
| Il-1β | –0.29 | 0.036 |
| Il-1RA | –0.23 | 0.094 |
| Il-1α | –0.14 | 0.306 |
| MIP-1β | –0.30 | 0.028 |
| MIP-1α | –0.10 | 0.485 |
| MCP-1 | –0.24 | 0.073 |
| Il-7 | –0.11 | 0.422 |
| IP-10 | –0.08 | 0.534 |
| **%DPA** | | |
| G-CSF | –0.32 | 0.017 |
| IL-6 | –0.30 | 0.029 |
| TNF-α | –0.28 | 0.038 |
| IL-8 | –0.21 | 0.121 |
| Il-1β | –0.23 | 0.099 |
| Il-1RA | –0.19 | 0.174 |
| Il-1α | –0.01 | 0.957 |
| MIP-1β | –0.29 | 0.030 |
| MIP-1α | 0.00 | 0.994 |
| MCP-1 | –0.29 | 0.030 |
| Il-7 | –0.12 | 0.381 |
| IP-10 | –0.09 | 0.492 |
| **%MUFA** | | |
| Eotaxin-1 | –0.16 | 0.245 |
| G-CSF | –0.03 | 0.804 |
| IL-6 | –0.19 | 0.174 |
| TNF-α | –0.06 | 0.656 |
| IL-8 | –0.05 | 0.715 |
| MCP-1 | –0.15 | 0.268 |
| Il-1β | –0.30 | 0.027 |
| Il-1RA | 0.04 | 0.789 |
| Il-1α | –0.15 | 0.281 |
| MIP-1β | –0.12 | 0.374 |
| MIP-1α | –0.09 | 0.511 |
| Il-7 | –0.06 | 0.656 |
| IP-10 | –0.13 | 0.336 |
| **%DHA** | | |
| Eotaxin-1 | –0.01 | 0.923 |
| G-CSF | –0.07 | 0.631 |
| IL-6 | –0.05 | 0.720 |
| TNF-α | –0.02 | 0.892 |
| IL-8 | 0.05 | 0.733 |
| MCP-1 | 0.00 | 0.974 |
| Il-1α | 0.02 | 0.893 |
| Il-1RA | 0.02 | 0.908 |
| Il-1α | 0.18 | 0.191 |
| MIP-1β | 0.04 | 0.791 |
| MIP-1α | 0.25 | 0.074 |
| Il-7 | –0.07 | 0.632 |
| IP-10 | 0.12 | 0.379 |
| **%AA** | | |
| Eotaxin-1 | –0.34 | 0.812 |
| G-CSF | 0.02 | 0.889 |
| IL-6 | –0.01 | 0.950 |
| TNF-α | 0.02 | 0.909 |
| IL-8 | –0.15 | 0.256 |
| MCP-1 | 0.01 | 0.960 |
| Il-1β | –0.12 | 0.391 |
| Il-1RA | –0.15 | 0.269 |
| Il-1α | –0.12 | 0.377 |
| MIP-1β | 0.00 | 0.985 |
| MIP-1α | –0.19 | 0.173 |
| Il-7 | 0.02 | 0.912 |
| IP-10 | –0.06 | 0.645 |
| **%OA** | | |
| Eotaxin-1 | –0.09 | 0.711 |
| G-CSF | –0.15 | 0.560 |
| IL-6 | –0.08 | 0.732 |
| TNF-α | –0.14 | 0.581 |
| IL-8 | –0.25 | 0.343 |
| MCP-1 | 0.09 | 0.715 |
| Il-1β | –0.11 | 0.674 |
| Il-1RA | –0.15 | 0.519 |
| Il-1α | 0.22 | 0.367 |
| MIP-1β | 0.34 | 0.156 |
| MIP-1α | 0.18 | 0.506 |
| Il-7 | 0.03 | 0.898 |
| IP-10 | 0.29 | 0.220 |
| **%Linoleic Acid** | | |
| Eotaxin-1 | –0.12 | 0.394 |
| G-CSF | –0.13 | 0.323 |
| IL-6 | –0.01 | 0.964 |
| TNF-α | –0.06 | 0.679 |
| IL-8 | 0.01 | 0.922 |
| MCP-1 | 0.14 | 0.297 |
| Il-1β | 0.05 | 0.741 |
| Il-1RA | 0.05 | 0.737 |
| Il-1α | 0.05 | 0.735 |
| MIP-1β | –0.01 | 0.964 |
| MIP-1α | –0.09 | 0.510 |
| Il-7 | 0.12 | 0.374 |
| IP-10 | 0.08 | 0.543 |
| **%n-3**  0.11  0.675  -0.25  0.076 | | |
| Eotaxin-1 | –0.25 | 0.062 |
| G-CSF | –0.24 | 0.080 |
| IL-6 | –0.24 | 0.083 |
| TNF-α | –0.18 | 0.182 |
| IL-8 | –0.22 | 0.101 |
| MCP-1 | –0.19 | 0.176 |
| Il-1β | –0.15 | 0.274 |
| Il-1RA | 0.02 | 0.908 |
| Il-1α | –0.20 | 0.144 |
| MIP-1β | 0.05 | 0.707 |
| MIP-1α | –0.11 | 0.425 |
| Il-7 | –0.06 | 0.635 |
| IP-10 | –0.06 | 0.668 |
| **%n-6** | | |
| Eotaxin-1 | –0.24 | 0.086 |
| G-CSF | –0.27 | 0.044 |
| IL-6 | –0.13 | 0.349 |
| TNF-α | –0.12 | 0.388 |
| IL-8 | –0.25 | 0.057 |
| MCP-1 | 0.02 | 0.858 |
| Il-1β | –0.02 | 0.860 |
| Il-1RA | –0.16 | 0.227 |
| Il-1α | –0.01 | 0.949 |
| MIP-1β | –0.14 | 0.294 |
| MIP-1α | –0.22 | 0.120 |
| Il-7 | 0.07 | 0.627 |
| IP-10 | –0.09 | 0.486 |
| **n-3/n-6** | | |
| Eotaxin-1 | –0.21 | 0.138 |
| G-CSF | –0.20 | 0.134 |
| IL-6 | –0.22 | 0.112 |
| TNF-α | –0.21 | 0.118 |
| IL-8 | –0.14 | 0.313 |
| MCP-1 | –0.20 | 0.137 |
| Il-1β | –0.17 | 0.217 |
| Il-1RA | –0.11 | 0.428 |
| Il-1α | –0.01 | 0.961 |
| MIP-1β | –0.18 | 0.194 |
| MIP-1α | 0.09 | 0.547 |
| Il-7 | –0.15 | 0.296 |
| IP-10 | –0.03 | 0.838 |
| **EPA+DHA/AA** | | |
| Eotaxin-1 | –0.14 | 0.314 |
| G-CSF | –0.23 | 0.087 |
| IL-6 | –0.20 | 0.161 |
| TNF-α | –0.19 | 0.154 |
| IL-8 | –0.02 | 0.881 |
| MCP-1 | –0.09 | 0.495 |
| Il-1β | –0.07 | 0.641 |
| Il-1RA | –0.05 | 0.698 |
| Il-1α | 0.07 | 0.594 |
| MIP-1β | –0.09 | 0.536 |
| MIP-1α | 0.18 | 0.192 |
| Il-7 | –0.18 | 0.207 |
| IP-10 | 0.04 | 0.779 |
| Percent serum fatty acid and tissue cytokine levels were analyzed by Spearman correlation. Presented is the Spearman correlation coefficient (r) and associated *P* value. Significant associations can be found in Figs. 2 and 3. | | |
